# Postoperative Diabetes Insipidus Mimicking Radiological Findings and Symptoms of Intracranial Hypotension: A Case Report

**DOI:** 10.7759/cureus.40398

**Published:** 2023-06-14

**Authors:** Muhammad A Kamal, David E Henshall, Mark A Hughes

**Affiliations:** 1 Department of Neurosurgery, Royal Infirmary of Edinburgh, Edinburgh, GBR; 2 Deanery of Clinical Sciences, College of Medicine & Veterinary Medicine, The University of Edinburgh, Edinburgh, GBR

**Keywords:** triphasic response, transsphenoidal surgery, cerebrospinal fluid (csf), neurosurgery, case report, cranial neurosurgery, dehydration, intracranial hypotension, diabetes insipidus, craniopharyngioma

## Abstract

Endocrine disturbances such as diabetes insipidus (DI) and syndrome of inappropriate antidiuretic hormone secretion (SIADH) are recognized complications of craniopharyngioma surgery, which occur due to damage to structures that produce or store antidiuretic hormone (ADH). Intracranial hypotension is a clinical syndrome that presents with headache and typical radiological features and can occur due to a leak of cerebral spinal fluid (CSF) in operations that involve the opening of the arachnoid (e.g., craniopharyngioma surgery).

We describe a patient presenting with headache, radiological evidence of intracranial hypotension, and chronic DI after craniopharyngioma surgery. This occurred in the absence of evidence of a CSF leak. The headache and radiological findings resolved after the identification and treatment of DI. Intracranial hypotension may have occurred secondary to dehydration in chronic DI.

A 48-year-old woman presented with progressive visual field loss due to cystic recurrence of a craniopharyngioma. She underwent redo (second) extended endoscopic transsphenoidal surgery, having previously undergone an uncomplicated debulking procedure two years prior. Her redo operation was uneventful, and her vision improved postoperatively. A lumbar drain was placed preoperatively to protect the skull base repair and was removed after 48 hours. In the initial postoperative period, she developed a clinical (polyuria) and biochemical picture consistent with DI, subsequently reverting to a SIADH, after which fluid and sodium homeostasis appeared to normalize, and she was discharged.

Two months after discharge, she re-presented with new headaches eased by lying flat. Magnetic resonance imaging (MRI) brain showed bilateral convexity subdural effusions and diffuse pachymeningeal enhancement, suggesting intracranial hypotension and raising concern for postoperative CSF leak. MRI spine did not show a CSF fistula at the site of the previous lumbar drain. Transsphenoidal examination under anesthesia showed a well-healed skull base repair and no evidence of CSF leak. She concurrently reported polyuria and polydipsia. A formal water deprivation test confirmed central DI. Treatment with desmopressin improved her headache, and a follow-up MRI brain showed resolution of the previous stigmata of intracranial hypotension.

This case report reminds physicians and neurosurgeons that systemic disorders (such as dehydration) can cause intracranial hypotension.

## Introduction

Endocrine disturbances such as diabetes insipidus (DI) and the syndrome of inappropriate antidiuretic hormone secretion (SIADH) are recognized complications of craniopharyngioma surgery [1]. These disturbances are relatively common before and after surgery and occur due to the proximity and involvement of the hypothalamus (that produces ADH) and posterior pituitary (that stores ADH).

Intracranial hypotension is a clinical syndrome that presents with a headache, which is classically orthostatic [2], and typical radiological features such as venous engorgement, brain sagging, subdural fluid collections, or hygromas [3]. Transsphenoidal surgery may be complicated by postoperative cerebrospinal fluid (CSF) leak [4], especially in operations that demand the opening of the arachnoid (as is necessary for the surgery of craniopharyngioma).

We describe a patient with a headache, radiological evidence of intracranial hypotension, and chronic postoperative DI. This occurred in the absence of evidence of a CSF leak. The headache and radiological findings resolved after the identification and treatment of DI. Intracranial hypotension may have occurred secondary to dehydration in chronic DI.

## Case presentation

In the context of progressive visual field loss, a 48-year-old woman underwent a second extended endoscopic transsphenoidal surgery for cystic recurrence of craniopharyngioma. Her index transsphenoidal operation two years prior had debulked the mass but not removed it entirely. Her other past medical history included pseudoxanthoma elasticum, hidradenitis suppurativa, and essential hypertension. She was on no regular medications, mobilized without any aids, lived independently with her family, and worked as an allied health professional.

Her redo (second) operation was uneventful, and she reported improvement in visual fields postoperatively (confirmed by perimetry). Due to the risk of CSF leak, a lumbar drain was inserted preoperatively to protect the skull base repair. There was no postoperative clinical concern for CSF rhinorrhea or leak, and the lumbar drain was removed 48 hours post operation. She was started on a maintenance dose of hydrocortisone postoperatively, as is our protocol following transsphenoidal surgery.

Early in the postoperative period, she was polyuric, and her sodium levels increased to 149 mmol/L, consistent with DI. She was treated with desmopressin for two days. After a brief period of stability, her sodium levels began to fall and desmopressin was stopped (serum sodium nadir of 123 mmol/L). Given the apparent development of SIADH, she was managed with one-liter fluid restriction, slow sodium tablets, and furosemide. Her sodium levels normalized, furosemide and sodium supplementation were stopped, and fluid restriction was relaxed entirely. After this point, fluid and sodium homeostasis appeared to have normalized (serum sodium of 141 mmol/L, normal fluid balance), and she was discharged on long-term hydrocortisone with endocrine and neurosurgery follow-up. Sodium levels were rechecked by her general (family medicine) practitioner one week later and found to be normal.

Two months later, she re-presented to her local emergency department with new headaches (frontal and occipital) that were eased slightly by lying flat, with nil improvement with codeine. She also reported intermittent fluid discharge from the nose. She appeared clinically euvolemic though she did report polydipsia and polyuria. Blood tests on admission under neurosurgery found serum sodium of 144 mmol/L, urea of 1.7 mmol/L, and creatinine of 60 μmol/L.

**Figure 1:**
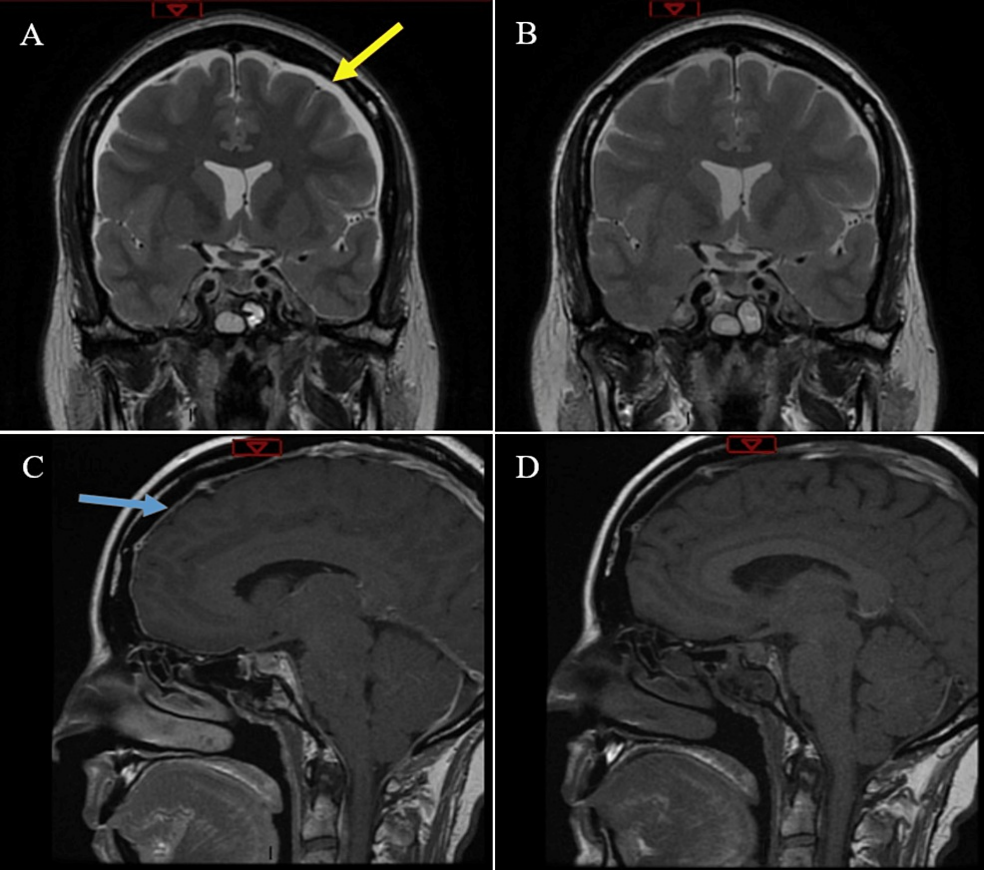
MRI brain demonstrating the presence and resolution of signs of intracranial hypotension Initial MRI demonstrating bilateral convexity subdural effusions (yellow arrow) with pachymeningeal enhancement (blue arrow) in coronal, T2-weighted (A) and sagittal, T1-weighted (C) images. Follow-up MRI three months later demonstrating resolution of above findings after the treatment of DI in coronal, T2-weighted (B) and sagittal, T1-weighted (D) images.

MRI brain demonstrated bilateral convexity subdural effusions and diffuse pachymeningeal enhancement, consistent with a chronic low-pressure state (Figure 1, Panels A and C). This, and the suggestion of rhinorrhea, raised concern for postoperative CSF leak. No CSF rhinorrhea was evident on examination, despite provocation maneuvers. The whole spine MRI did not suggest a CSF leak at the site of the prior lumbar drain or elsewhere. Transsphenoidal examination under anesthesia was performed. This illustrated a well-healed skull base repair and no sign of CSF leak, despite Valsalva maneuvers. Plasma osmolality was 301 (280-290 mOsmol/kg), and urine osmolality was 247 (50-1200 mOsmol/kg). A water deprivation test subsequently confirmed cranial DI, with urine osmolality increasing to 562 mOsmol/kg after 2 micrograms of desmopressin.

Following a diagnosis of DI, intranasal desmopressin was commenced. Due to her antidiuretic hormone (ADH) sensitivity, after titration, only a pediatric dose (5 micrograms at night) was required. Over the next fortnight, this resulted in significant improvement in her headaches, and she was soon fit for discharge. On day 26 of admission, she was discharged on (and now continues to take) 5 micrograms of desmopressin, with neurosurgical and endocrine follow-up. Follow-up MRI brain three months later (Figure 1, Panels B and D) demonstrated resolution of the bilateral convexity subdural effusions and prior pachymeningeal enhancement.

## Discussion

**Figure 2:**
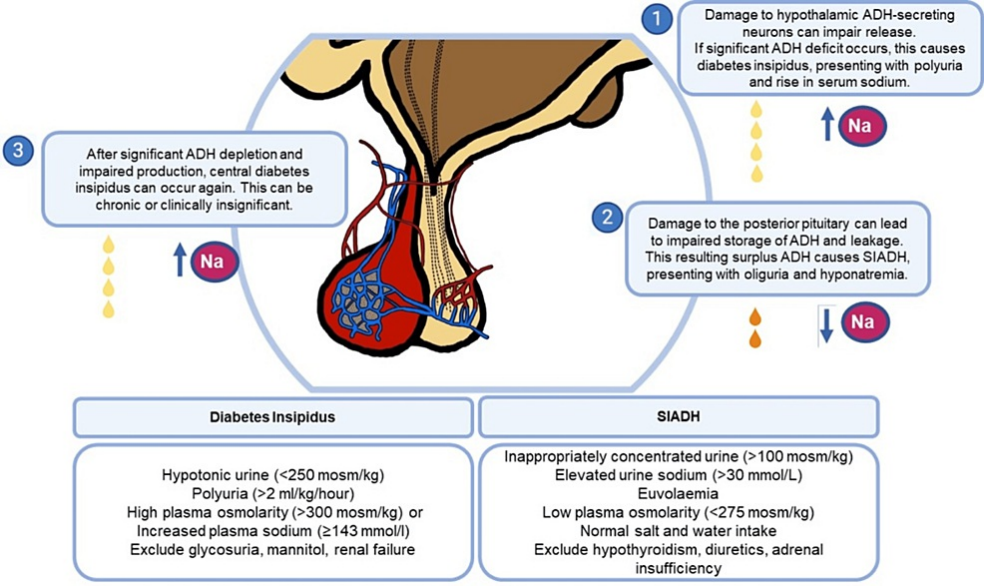
Disturbances of the hypothalamic-pituitary axis after transsphenoidal surgery including the triple-phase response ADH: Antidiuretic hormone; SIADH: Syndrome of inappropriate ADH secretion; Na: Sodium. Image credits: Authors of this study.

Postoperative central DI occurs in approximately 20% of patients after transsphenoidal surgery, with a greater risk associated with the resection of craniopharyngiomas compared to pituitary adenomas [5]. This patient exhibited the recognized triple-phase response with initial DI, subsequent SIADH, and then chronic DI (the mechanisms are explained in Figure 2).

Intracranial hypotension occurs due to low CSF pressure and classically presents with orthostatic headache (see Table 1 for diagnostic criteria) [2]. Our patient did not report classical postural descriptors; however, there was a clear temporal relationship between the resolution of the headache and radiological features.

**Table 1:** The diagnosis of intracranial hypotension. The diagnostic criteria for headache are associated with low cerebrospinal fluid pressure, characteristic clinical features of the headache, and radiological features of intracranial hypotension [2,3,6-8]. ICHD-3: The International Classification of Headache Disorders, 3rd edition.

Headache attributed to low cerebrospinal fluid (CSF) pressure: diagnostic criteria	Intracranial hypotension: clinical features	Intracranial hypotension: radiological features
A. Any headache, meeting C	Headache is usually orthostatic but not always	Diffuse pachymeningeal enhancement
B. With: (1) low cerebrospinal fluid (CSF) pressure (<60 mm CSF) or (2) evidence of CSF leakage on imaging	Usually associated with neck pain, tinnitus, changes in hearing, photophobia, and/or nausea	Diffuse dural enhancement of the spinal cord and/or abnormal intensity around the root sleeves
C. Developed in temporal relation to the low CSF pressure, CSF leakage or led to its discovery	Remits after normalization of CSF pressure or sealing of CSF leak	Subdural and/or spinal epidural fluid collections
D. Not better accounted for by another ICHD-3 diagnosis		Brain sagging
		Pituitary engorgement

**Figure 3:**
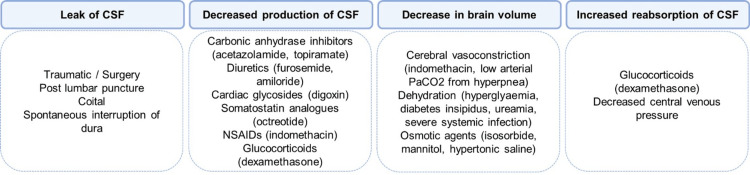
Mechanisms of reduced intracranial pressure Four suggested mechanisms of reduced intracranial pressure with associated causes [3,9-14]. NSAID: Nonsteroidal anti-inflammatory drug; PaCO_2_: Partial pressure of carbon dioxide.

Mechanisms for reduced CSF volume include CSF leak, reduced production, increased reabsorption, and a decrease in brain volume (Figure 3) [3]. In the absence of CSF leak on transsphenoidal examination under anesthesia and MRI of the brain and whole spine, intracranial hypotension in our patient may have occurred secondary to dehydration in the context of chronic DI.

In animal models, ADH-deficient rats have reduced basal CSF production in comparison with ADH-competent rats [15]. Furthermore, in response to osmotic dehydration (by infusion of hypertonic saline), they had comparatively reduced CSF production, demonstrating the importance of intact ADH signaling in the maintenance of CSF volume [15]. In healthy adult sheep, 48 hours of water deprivation did not affect CSF production or absorption. However, it did result in reduced intraventricular and central venous pressures [9]. In humans, dehydration can result in significant changes in brain volume, with shrinkage of brain tissue and enlargement of the ventricular system [10,11]. In our case, ADH insufficiency, plasma hyperosmolarity, or fluid losses may have altered CSF production, absorption, or volumes of brain tissue.

## Conclusions

We describe a patient re-presenting with a new headache and radiological evidence of intracranial hypotension after expanded endoscopic endonasal surgery for recurrent craniopharyngioma. Postoperative CSF leak was initially considered a likely cause, but there was no evidence of this in the clinical, radiological, or surgical investigation. Instead, the headache and radiological findings fully resolved after the identification and treatment of DI.

In this case, intracranial hypotension may have occurred secondary to dehydration in chronic DI. This case report reminds physicians and neurosurgeons that systemic disorders (such as dehydration) can cause intracranial hypotension.
